# Local Random Pattern Flap Coverage for Implant Exposure following Open Reduction Internal Fixation via Extensile Lateral Approach to the Calcaneus

**DOI:** 10.1186/s12891-021-04427-x

**Published:** 2021-06-21

**Authors:** Yingjie Liu, Peihua Cai, Liang Cheng, Yanfeng Li

**Affiliations:** Department of Orthopedics, Shanghai Jiao Tong University Affiliated Sixth People’s Hospital, 600 Yishan road, Shanghai, 200233 China

**Keywords:** Lateral approach calcaneus, Random pattern flap, Implant exposure

## Abstract

**Background:**

Skin necrosis and implant exposure most often appear at the corner of Extensile Lateral Approach for open reduction and internal fixation (ORIF) for displaced intra-articular fracture of the calcaneus. Flap transfer is often used for coverage of this implant exposure. We introduced a new simple local random pattern flap to cover the implant exposure.

**Methods:**

From March 2017 to March 2020, 12 patients with implant exposure after ORIF for displaced intra-articular fracture of the calcaneus were treated with this procedure. The sizes of the defects ranged from 2 × 2 cm^2^ to 5 × 2 cm^2^. A local random pattern flap was designed according to the defect size. The lower edge of the flap was along with the wound upper edge and extended distally. The upper horizontal incision of the flap was made at the lateral malleolus level with a length of 5–7 cm depending on the wound defect. Then the random pattern flap was elevated and transferred to cover the defect area.

**Results:**

The mean follow-up duration was 6.3 months (ranging 4–13 months). All 12 flaps were uneventfully healed and all patients were able to wear shoes, and no debulking procedures were required.

**Conclusion:**

The local random pattern flap could be a choice for surgeons when implant exposure at the corner of Extensile Lateral Approach to the Calcaneus occurs.

## Introduction

Calcaneal fracture comprises 1–2% of all fractures which is the most frequently fractured of all the tarsal bones [1]. 71% of calcaneal fractures are intra-articular calcaneal fractures, which are the most challenging types for treatment [2]. The management of Displaced intra-articular calcaneal fractures can be divided into four categories: non-operative management; open reduction and internal fixation (ORIF); minimally invasive reduction and fixation; primary ORIF and subtalar arthrodesis [2]. Open reduction and internal fixation (ORIF) for displaced intra-articular fracture of the calcaneus has been widely advocated by most experts, as it generally provides good to excellent functional outcomes and the ability to anatomically restore the subtalar joint [3, 4]. Several open Operative techniques have been described in the past, of which the extended lateral approach has been applied most frequently [5–8]. The rate of wound complications after ORIF of closed calcaneal fractures via the extensile lateral approach has ranged from 0 to 27% [5, 9]. The corner of Extensile Lateral Approach to the Calcaneus is the most likely site of wound healing complications, with skin necrosis. Skin necrosis and implant exposure most often appear in this area.

Flap transfer is often used for coverage of such implant exposure. Mueller et al reported that they used the musculocutaneous sural artery flap for coverage of implant exposure after calcaneal fracture [10]. However, there is often morbidity at the donor site and shoe-fitting problems because of a bulky contour. A free flap is also used to cover calcaneal soft-tissue defects [11], but the procedure is time-consuming and technically demanding. In this study, we used a local random skin flap for soft tissue coverage of implant exposure after calcaneal fracture procedures.

## Materials and Methods

A retrospective review was performed of patients with implant exposure after calcaneal fracture procedures from March 2017 to March 2020. This study was approved by the Institutional Review Board of Shanghai Jiao Tong University Affiliated Sixth People’s Hospital. 12 patients out of all 22 patients with implant exposure at the corner of the Extensile Lateral Approach to the Calcaneus were included in this study. The exclusion criteria: the positive culture result of wound specimen collection before operation was excluded.

Seven of the patients were men and five were women with a mean age of 44 years (range, 32–59 years). The sizes of the defects ranged from 2 × 2 cm^2^ to 5 × 2 cm^2^. The details of the included cases are listed in Table 1. Six of the patients were smokers and they were asked to stop smoking before the operation. Blood glucose levels were kept under 10.0 mmol/L in the four diabetic patients by injecting insulin before the operation.

**Table 1:** Patient demographics and outcomes

Patient	Age (years)	Sex	Defect size (cm^2^)	NPD	Smoker	Diabetes	Flap survival	ROM
1	32	M	3 × 2	Yes	Yes	No	Complete	Fully
2	37	F	2 × 2	No	No	No	Complete	Fully
3	43	M	2 × 2	No	Yes	Yes	Complete	Fully
4	50	M	5 × 2	Yes	No	No	Complete	Slightly reduced
5	51	F	4 × 2	Yes	No	Yes	Complete	Slightly reduced
6	59	M	3 × 2	Yes	Yes	No	Complete	Fully
7	41	F	2 × 2	No	No	No	Complete	Fully
8	43	M	4 × 2	Yes	Yes	Yes	Complete	Slightly reduced
9	35	M	2 × 2	No	Yes	No	Complete	Fully
10	54	F	3 × 2	Yes	No	Yes	Complete	Fully
11	49	F	3 × 2	Yes	No	No	Complete	Fully
12	33	M	2 × 2	No	Yes	No	Complete	Fully

### Operative Technique

**Fig. 1:**
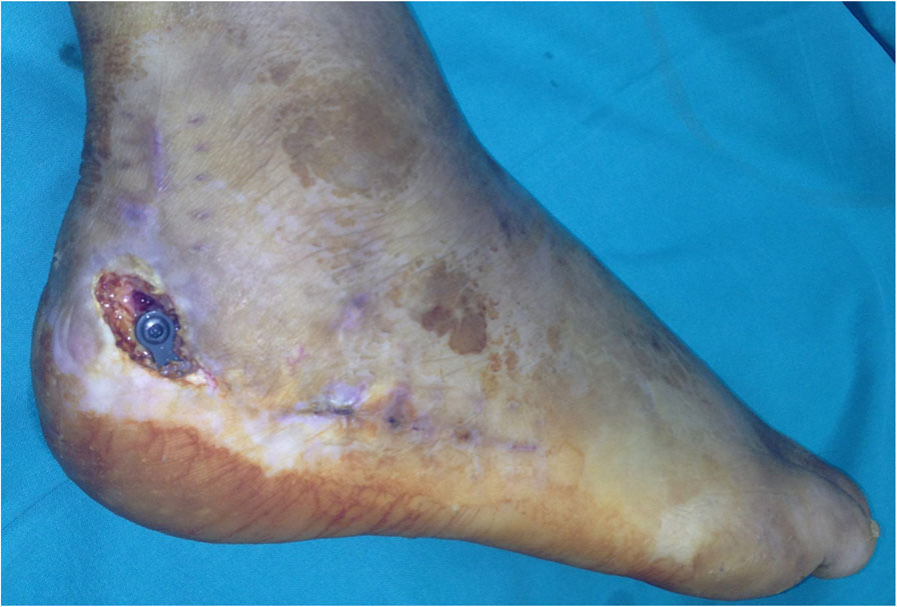
Skin necrosis after ORIF for displaced intra-articular fracture of the calcaneus. There is a skin defect over the hardware

**Fig. 2:**
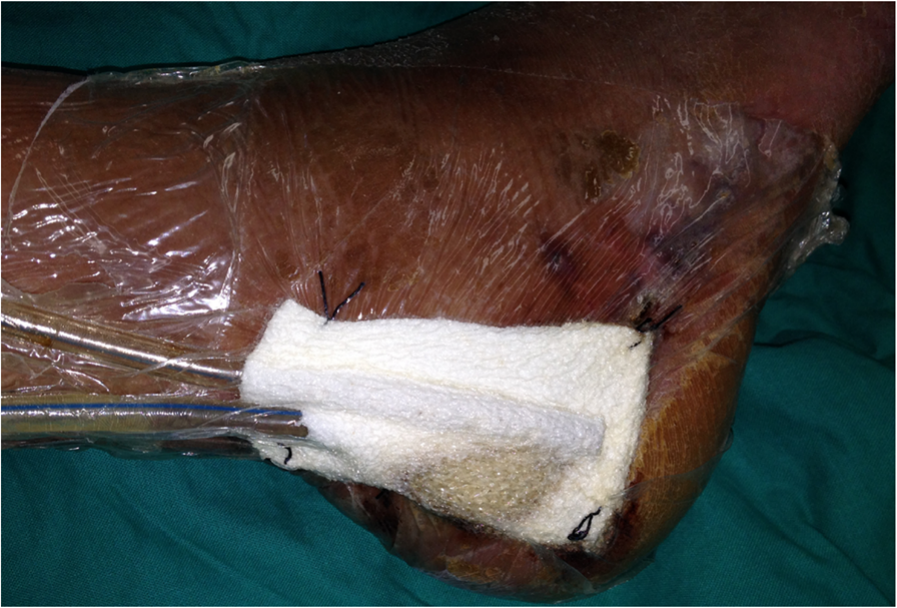
After wound debridement, a negative-pressure dressing was applied to wound

A debridement and a negative-pressure dressing were applied for 3–5 days. Then the wound specimens were collected for microbial cultures and the wounds were covered by a negative-pressure dressing. When the cultures of wound specimen collection were negative, the local random flap was used to cover the wounds (Figs. 1 and 2).

**Fig. 3:**
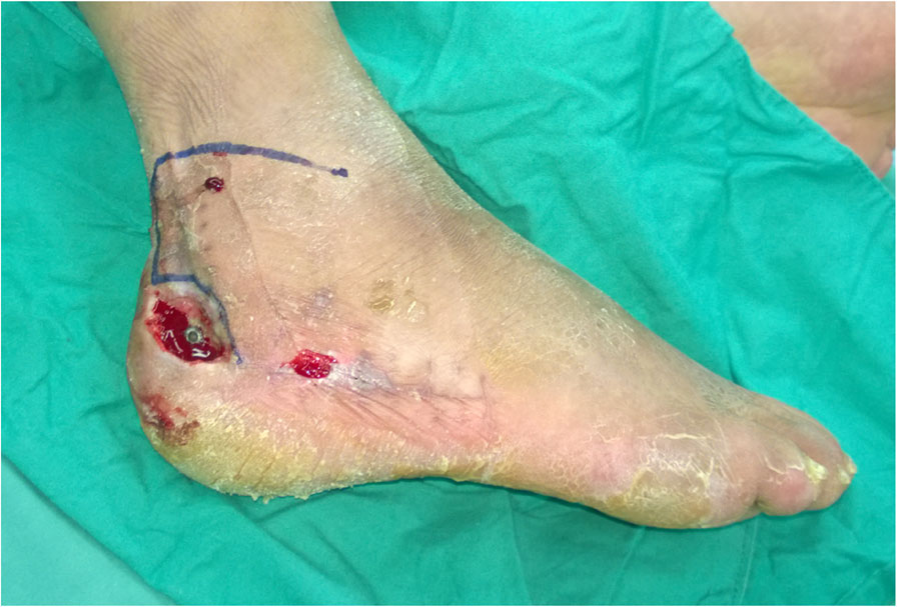
The design of the local random pattern flap

**Fig. 4:**
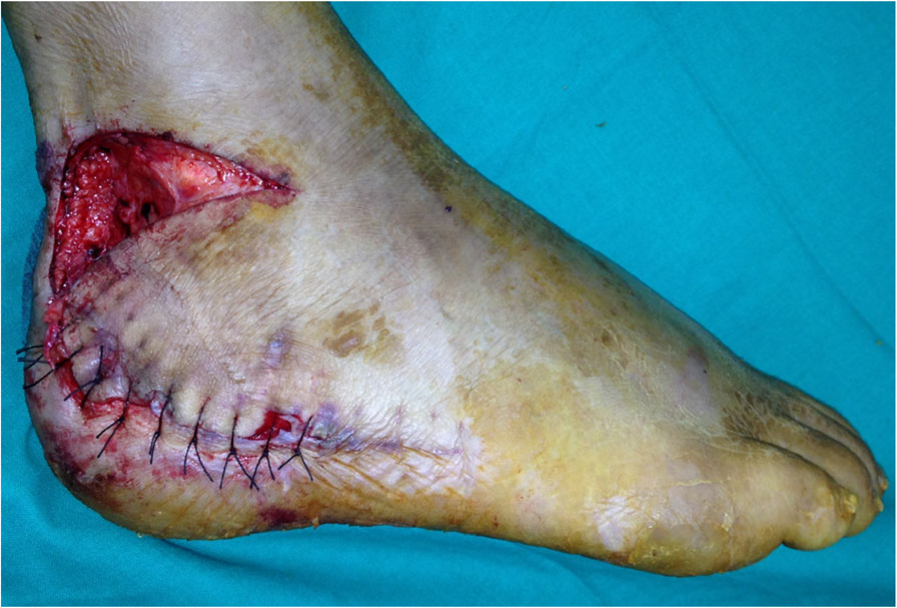
A local random pattern flap was transferred to cover the skin defect

**Fig. 5:**
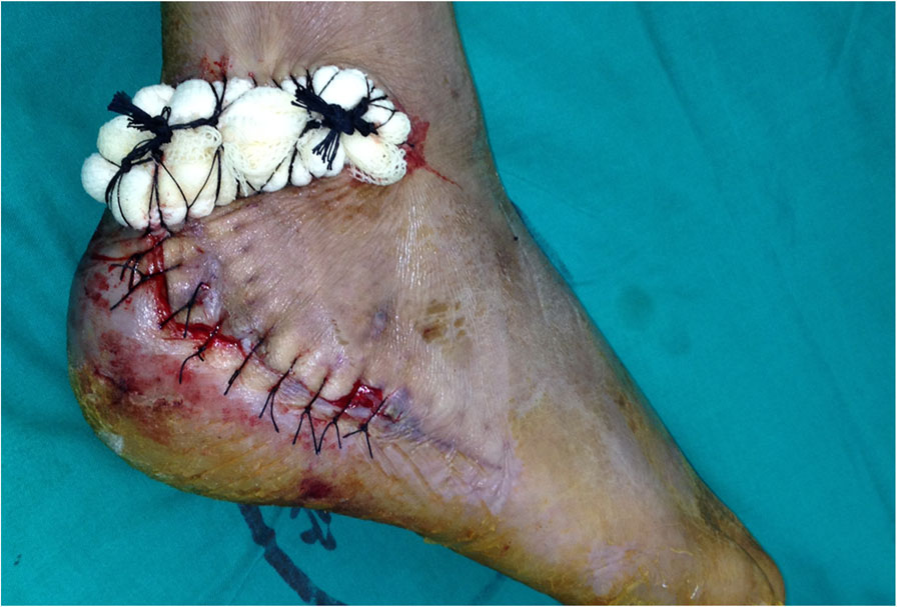
The donor site was covered by split-thickness skin graft

Patients were placed in a lateral position for the local random pattern flap procedure. A local random pattern flap was designed according to the defect size. The lower edge of the flap was along with the wound upper edge and extended distally. The upper horizontal incision of the flap was made at the lateral malleolus level with a length of 5–7 cm depending on the wound defect (Fig. 3). The lesser saphenous vein and the sural nerve were dissected and protected carefully. Then the random pattern flap was elevated and transferred to cover the defect area. The donor site was covered by a split-thickness skin graft (Figs. 4 and 5). The mean operative time was 40–60 min.

### Postoperative Treatment

The patients remained in bed for 2 weeks, avoiding any pressure on the flap by suspending the leg on an external device. Low molecular weight dextran was given to improve the micro-circulation, and antibiotics were administered for 48 h to prevented infection. The six smoking patients were asked to stop smoking. Blood glucose levels were maintained below 10.0 mmol/L in the four diabetic patients.

## Results

**Fig. 6:**
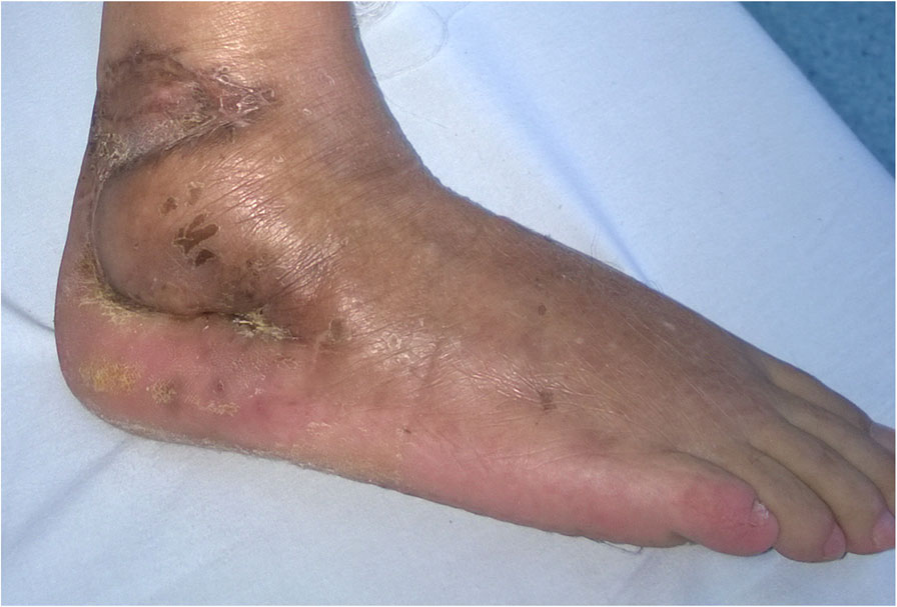
Flap healed uneventfully with good functional and aesthetic result

The mean follow-up duration was 6.3 months (range 4–13 months). All the patients were satisfied with the functional and aesthetic results (Table 1). During the follow-up period, all patients were able to wear shoes, and no debulking procedures were required (Fig. 6). The ankle range of motion was preserved in nine patients, and was slightly reduced in three patients. However, all patients were able to walk without walking aids.

## Discussion

The extended lateral approach is widely applied for intra-articular calcaneal fracture ORIF and offers advantages of achieving adequate fracture reduction with the risk of wound-healing complications and infection [12]. An L-shaped lateral incision is made to expose the calcaneus, but the corner of the incision is the most likely site of wound healing complications, such as necrosis. Skin necrosis, wound dehiscence, and plate exposure most often appear in this area, because there is less subcutaneous tissue under the lateral calcaneal flap. Blood flow to the lateral calcaneal flap is primarily supplied by the lateral calcaneal artery (LCA), and complications in wound healing, such as ischemia of the lateral calcaneal flap, can arise from damage to the LCA. Borrelli et al. concluded that, based on the position of the LCA, it is vulnerable to injury caused by the vertical incision in the lateral approach [13].

Smoking has a detrimental effect on the healing of wounds. Smoking causes blood vessels to contract, thus reducing blood supply to the extremities, with decreases in the contractility of vessels, blood flow rate, and efficiency of oxygen transportation. These negative effects are reversible [14]. The literature generally confirms that nicotine abstinence for 4–8 weeks preoperatively is advantageous and post-operative complications may be reduced if patients refrain from smoking for 10 days after surgery [15, 16]. In our study, the six smoking patients were asked to stop smoking before the operation and to abstain for another 2 weeks postoperatively. The flaps in all six patients healed uneventfully.

Diabetes was considered as a significant independent risk factor for wound complications [17]. Diabetes can impede wound healing and predispose patients to infection through ischemia secondary to microvascular abnormality. In this study wound complication after ORIF occurred in all four diabetic patients. During the flap operation period, the blood glucose levels were maintained below 10.0 mmol/L in all four diabetic patients by insulin injection. Finally, the flap survived.

When skin flap necrosis occurs at the corner of the Extensile Lateral Approach , local wound care, debridement, changing dressings, antibiotics and skin flap transplantation should be attempted, in that order, with a favourable outcome being largely dependent on early diagnosis and treatment [18]. Herscovici et al. reported that debridement of all necrotic tissue and changing dressings once a day using thick povidone iodine gauze often resulted in a good outcome [19]. However, if the necrotic area results in implant exposure, skin flap transplantation might be needed. A reversed sural nerve flap is most frequently used because of its rich blood supply and the simplicity of the operation [20]. The most common complication encountered with the reversed sural type of flap is venous congestion which may result in partial- or full- thickness flap necrosis [21]. Transfer of a free flap has also been reported to cover a defect in this area [11]. This technique requires specialized microsurgical training and is best performed in patients who are able to withstand prolonged general anesthesia [22]. There is also morbidity at the donor site and later shoe-fitting problems because of a bulky contour. The abductor digiti minimi (ADM) flap can be used for small defects at the corner of the Extensile Lateral Approach. CL Wang et al reported that they used the abductor digiti minimi muscle flap as a muscular plug between the wound and the plate after ORIF of calcaneus fractures [23] . However, loss of the ADM muscle may result in lack of plantar lateral padding, which may result in discomfort. In this study, we reported a local random pattern flap for coverage of the skin necrotic area with implant exposure. Local random pattern flaps are flaps whose success depends on a length-to-width ratio (1.5:1) with no specific blood flow at their base. Attinger et al. used a local muscle flap for the treatment of hardware exposure [24]. In our cases, we used a local random pattern flap to cover the hardware exposure. The benefits of this flap include a similar tissue composite of the hardware exposure area, no specialized microsurgical training, and easy for surgeons to operate.

However, our technique has a limitation. Before applying the local random flap for hardware exposure, we needed to collect some wound specimen for culturing to indentify pathogens. If the result was positive which implied that there was a deep infection at the surgery site, the local random flap was not recommended. Thus, in this study, the wound specimen was collected and cultured to indentify pathogens before operations. The local random flap was applied to the patients with negative results.

## Conclusion

Our study is the first report of the simple flap, a local random pattern flap, for the coverage of implant exposure after ORIF for calcaneal fractures. All 12 flaps were uneventfully healed. The random pattern flap probably survived through perfusion from a perforator from the anastomotic arcade around the ankle. However, the reliability of the local random pattern flap needs to be further evaluated with a larger number of patients. The technique under discussion will need a larger and preferably multicentric study to finally assess the reliability of the technique. Nevertheless, the flap procedure is easy to perform and needs no microsurgery skills. It could be an option for surgeons when implant exposure occurs at the corner of Extensile Lateral Approach.

## Data Availability

The datasets generated during and analyzed during the current study are not publicly available due to the privacy policy but are available from the corresponding author on reasonable request.
